# The relationship between early atherosclerosis and endothelial dysfunction in type 1 diabetic patients as evidenced by measurement of carotid intima-media thickness and soluble CD146 levels: a cross sectional study

**DOI:** 10.1186/1475-2840-12-153

**Published:** 2013-10-18

**Authors:** Sema Ciftci Dogansen, Aysen Helvaci, Mine Adas, Suzan Deniz Onal

**Affiliations:** 1 Internal Medicine Clinic, Okmeydani Training and Research Hospital, Istanbul, Turkey; 2 Department of Cardiology, Internal Medicine Clinic, Okmeydani Training and Research Hospital, Istanbul, Turkey; 3 Department of Endocrinology, Internal Medicine Clinic, Okmeydani Training and Research Hospital, Istanbul, Turkey; 4 Department of Radiology, Okmeydani Training and Research Hospital, Istanbul, Turkey; 5 Present address: Department of Endocrinology and Metabolic Diseases, Canakkale State Hospital, Canakkale, Turkey

**Keywords:** Atherosclerosis, CIMT, Endothelial damage, sCD146, Type 1 diabetes

## Abstract

**Background:**

Detection of early vascular changes prior to clinical manifestations of atherosclerosis, such as increased arterial carotid intima-media thickness (CIMT) and impaired endothelial function is of paramount importance for early identification of subjects at increased risk of accelerated atherosclerosis. The present study was designed to evaluate the relationship between early atherosclerosis and endothelial dysfunction in type 1 diabetic patients based on measurements of CIMT and soluble CD146 (sCD146) levels.

**Methods:**

Thirty-seven patients with type 1 diabetes, 14 males (37.8%) and 23 females (62.2%), of mean (SD) age 26.2 (4.1) years admitted to the outpatient diabetes clinic at Okmeydani Training and Research Hospital, Istanbul, between January 2008 and December 2012, and 37 healthy controls, 16 males (43.2%) and 21 females (56.8%), of mean (SD) age 25.8 (3.1) years, selected from relatives of patients, were included. Anthropometric measures; fasting plasma glucose; and serum HbA1c, total cholesterol, HDL-cholesterol, LDL-cholesterol, triglyceride and creatinine concentrations were compared, as were CIMT and serum sCD146.

**Results:**

Mean (SD) sCD146 levels were significantly higher in patients than in controls (314.6 (141.9) ng/ml vs. 207.8 (34.5) ng/ml, p = 0.001), but mean (SD) CIMT did not differ (0.5 (0.1) mm vs. 0.4 (0.1) mm). ROC curves for sCD146 significantly differed in differentiating type 1 diabetics from healthy controls (p = 0.0047) with a significantly higher percentage of patients than controls having sCD146 levels >260 ng/ml (21/37 (56.8%) vs. 2/37 (5.4%), p = 0.00011).

**Conclusion:**

Our findings emphasize that sCD146 levels may be a more sensitive marker than CIMT for earlier identification of type 1 diabetic patients at high risk for atherosclerosis.

## Introduction

Atherosclerosis is an important macrovascular complication and the major cause of morbidity and mortality in patients with diabetes mellitus [1]. Moreover, diabetes mellitus itself is a risk factor for atherosclerosis [2]. Although type 1 diabetics are at lower risk for atherosclerotic cardiovascular disease than type 2 diabetics due to the younger age of the former group the relative risk is 10 times higher in type 1 diabetics than in non-diabetics of similar age [3-5]. In addition, type 1 diabetes has been associated with increased intima media thickness and decreased endothelial function even in childhood [6], emphasizing the importance of early detection and prevention of macrovascular disease in patients with juvenile-onset type 1 diabetes.

Owing to its central role in vascular homeostasis, endothelial function and endothelial cells themselves may play a key role in vascular diseases related to atherosclerosis [7]. In addition, endothelial dysfunction has been considered the critical mechanism underlying the atherosclerotic pathogenesis of diabetes [8]. Measuring biological markers of vascular endothelial function in vivo may therefore provide insights into the evolution and prognosis of vascular diseases [7]. For example, although circulating endothelial cells (CECs) expressing the membrane glycoprotein CD146 are rarely found in the blood of healthy subjects, their release from damaged endothelium in individuals with cardiovascular and inflammatory diseases leads to increased levels of CD146 in the peripheral circulation [7,9].

Although there is no standardized method for detecting CECs, measurement of soluble CD146 (sCD146) has become the most commonly used specific marker for the detection of CECs in peripheral blood [10]. sCD146, also known as S-Endo 1-associated antigen, MelCAM and MUC, is located at intercellular margins and therefore is likely to act as an adhesion molecule [10-12].

Another established clinical index of vascular endothelial function closely related to atherosclerosis is carotid intima-media thickness (CIMT) [13]. Measurements of CIMT have been considered relatively simple and cost-effective, as well as being a surrogate marker for subclinical atherosclerosis [14].

The detection of early vascular changes prior to the clinical manifestations of atherosclerosis, such as increased CIMT and impaired endothelial function, are of paramount importance for the early identification of subjects at increased risk of accelerated atherosclerosis [15,16]. However, although both parameters are commonly measured in clinical trials, no consensus has yet been reached on the superiority and/or priority of morphological vs. endothelial alterations in subjects at increased risk for atherosclerosis [16]. Furthermore, although several studies have reported that CIMT can predict subclinical atherosclerosis in diabetic patients [17-22], less is known on whether CECs can act as a marker of endothelial cell activation and damage in diabetic individuals [23-27].

This study was therefore designed to evaluate sCD146 levels and CIMT as early predictors of endothelial damage and atherosclerosis, respectively, in type 1 diabetic patients without diabetes-related complications or additional cardiovascular risk factors and to compare CIMT and sCD146 levels in type 1 diabetics and healthy controls.

## Methods

### Study population

Thirty-seven patients with type 1 diabetes, 14 males (37.8%) and 23 females (62.2%), of mean (SD) age 26.2 (4.1) years admitted to the outpatient diabetes clinic at Okmeydani Training and Research Hospital, Istanbul, between January 2008 and December 2012, and 37 healthy controls, 16 males (43.2%) and 21 females (56.8%), of mean (SD) age 25.8 (3.1) years, selected from relatives of patients, were included. Patients were included if they were aged 20–40 years, had type 1 diabetes for at least one year, were receiving only insulin as anti-diabetic treatment, and lacked diabetes related complications and additional cardiovascular risk factors. Controls were included if they were aged ≥20 years and had normal fasting blood glucose concentrations. Individuals were excluded if they had any systemic disease known to cause endothelial dysfunction, including systemic hypertension (defined according to the JNC-7 criteria) [28], hyperlipidemia (defined according to the NCEP-ATP3 criteria) [29], coronary artery disease, peripheral vascular disease, retinopathy, neuropathy, carotid artery disease, inflammatory or infectious processes within the last 3 months, or malignancy. Individuals were also excluded if they had a body mass index (BMI) >25 kg/m^2^, had undergone any invasive procedure in the month before enrollment, had abnormal renal (including micro-albuminuria) or hepatic biochemical markers, were receiving any medication affecting endothelial function, or were active smokers. Written informed consent was obtained from each subject following a detailed explanation of the objectives and protocol of the study, which was conducted in accordance with the ethical principles, stated in the “Declaration of Helsinki” and was approved by the Okmeydani Training and Research Hospital Ethics Committee.

### Assessments

Anthropometric measures, fasting plasma glucose and HbA1c concentrations, and serum total cholesterol, HDL-cholesterol, LDL-cholesterol, triglyceride, creatinine, and sCD146 levels were measured, as was CIMT.

### Determination of sCD146

Blood samples were collected in tubes containing EDTA and centrifuged at 3000 rpm at 4°C for 15 min. The supernatants were decanted and frozen at -80°C until assayed. sCD146 levels were assayed using CY-QUANT ELISA sCD146 test kits (Biocytex, France). These assays are based on a plastic support coated with specific mouse monoclonal anti human CD146 F(ab’)2 fragments (Reagent 1), which bind to sCD146, and peroxidase-coupled mouse monoclonal anti CD146 antibody (Reagent 5), which binds to a remaining free antigenic determinant of CD146. The bound peroxidase is reacted with TMB substrate (Reagent 6) for a predetermined time. The reaction is stopped (Reagent 8), and the intensity of the signal is directly proportional to the sCD146 level of the original sample, with sCD146 levels determined from standard curves [30].

### Measurement of CIMT

CIMT in both diabetic and healthy individuals was evaluated by combined B-mode and color Doppler ultrasonography (Siemens, Germany), using a sectorial probe of 7.5 MHz with axial and lateral resolution of 0.15 mm. CIMTs were measured using a predetermined, standardized scanning protocol for the right and left carotid arteries. The proximal part of the carotid bulb was identified on both sides, and the segments of the common carotid arteries 3 cm proximal to the bulb were scanned. Measurements were performed on plaque-free segments by the same expert under single-blind conditions. A segment with an IMT >1.0 mm was defined as thickened, whereas IMT ≤1.0 mm was considered normal.

### Statistical analysis

Calculation of group size showed that at least 37 patients per group were needed to detect a correlation coefficient of 0.46 between sCD146 and CIMT values in patients with type I diabetes mellitus at a statistical power (1 minus the β value) of 90% allowing for a type I (α) error of 0.05.

All statistical analyses were performed using SPSS version 15.0 software (SPSS Inc. Chicago, IL, USA). Categorical data were compared using chi-square (χ^2^) tests. Student’s t tests and Mann Whitney U tests were used to compare normally and non-normally distributed numerical variables, respectively. Receiver operating characteristic (ROC) curves for sCD146 and CMIT were generated and the area under the curve (AUC) was calculated to determine the cutoffs for each yielding optimal sensitivity, specificity, positive predictive value (PPV), negative predictive value (NPV) and likelihood ratio (LR). Data were expressed as “mean (standard deviation; SD)”, minimum-maximum and percent (%) where appropriate. p < 0.05 was considered statistically significant.

## Results

### Demographic characteristics, anthropometric measurements and blood biochemistry

Patients were determined to be diabetic for a mean (SD) 6.9 (3.3) years. Patient and control groups were similar in mean (SD) age, mean (SD) BMI, and gender distribution. Apart from significantly higher fasting plasma glucose and HbA1c concentrations in patients than in controls (p = 0.0001 each), there were no significant between group differences in blood biochemistry (Table 1).

**Table 1:** Demographic characteristics, BMI and blood biochemistry in the patient and control groups

	**Type 1 diabetes**	**Healthy controls**	**p value**
	**(n = 37)**	**(n = 37)**	
	**Mean (SD)**		
Age (years)	26.16 (4.07)	25.84 (3.05)	0.699
Gender (F/M)	23/14	21/16	0.636
Body mass index (kg/m^2^)	22.43 (2.26)	21.41 (2.29)	0.057
Fasting plasma glucose (mg/dL)	119.27 (27.40)	85.08 (7.33)	**0.0001**
HbA1c (%)	7.11 (0.9)	5.17 (0.25)	**0.0001**
Total cholesterol (mg/dL)	163.54 (20.33)	161.08 (16.33)	0.568
LDL cholesterol (mg/dL)	86.95 (9.12)	82.11 (10.82)	**0.041**
HDL cholesterol (mg/dL)	53.00 (12.30)	54.19 (10.20)	0.652
Triglycerides (mg/dl)	66.11 (21.55)	68.59 (8.99)	0.519
Creatinine (mg/dl)	0.8 (0.3)	0.8 (0.2)	0.221

Values in bold indicate statistical significance (p<0.05).

### sCD146 and CIMT values

Mean (SD) sCD146 levels were significantly higher in patients with type 1 diabetes than in control subjects (314.6 (141.9) ng/ml vs. 207.8 (34.5) ng/ml, p = 0.001). In contrast, mean (SD) CIMT was similar in the patient and control groups (0.5 (0.1) mm vs. 0.4 (0.1) mm) (Table 2).

**Table 2:** sCD146 and CIMT values in the patient and control groups

	**Type 1 diabetes**	**Healthy controls**	**p value**
	**(n = 37)**	**(n = 37)**	
	**Mean (SD)**		
**sCD146 (ng/ml)**	314.72 (139.88)	207.80 (34.51)	**0.001**
**CIMT (mm)**	0.48 (0.10)	0.44 (0.06)	0.153

CIMT Carotid artery intima-media thickness. Values in bold indicate statistical significance (p<0.05).

### ROC curves for sCD146 and CMIT to differentiate type 1 diabetes mellitus from healthy controls

**Figure 1:**
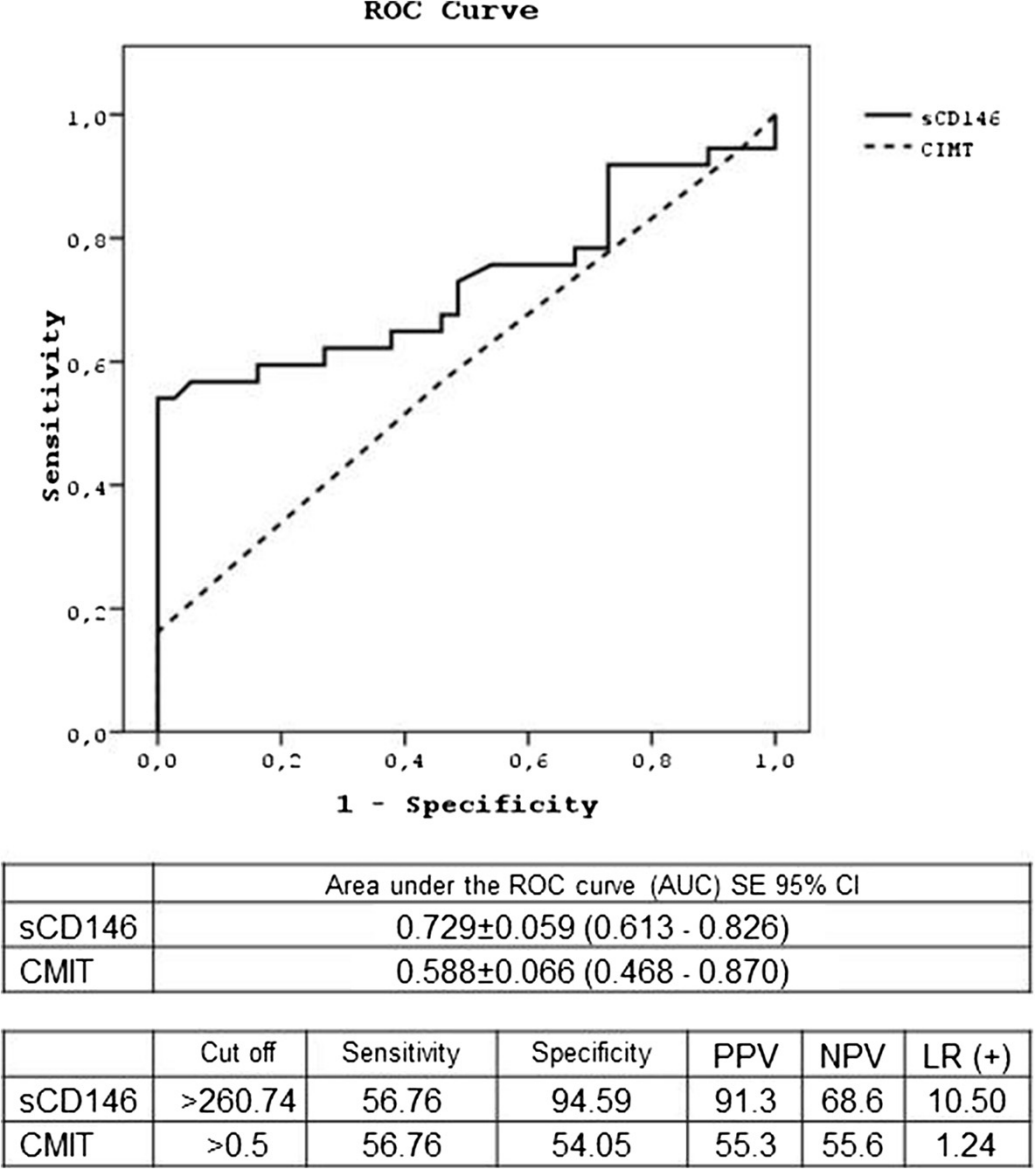
ROC curves for sCD146 and CMIT differentiating subjects with type 1 diabetes mellitus from healthy controls.

ROC analysis showed that an sCD146 level of >260.74 ng/ml was the optimum cutoff for differentiating individuals with type 1 diabetes mellitus and healthy controls, with a sensitivity of 56.8%, a specificity of 94.6% and an LR of 10.5%. ROC analysis also showed that the most suitable cut-off value for CIMT was >0.5 mm, with a sensitivity, specificity and LR of 56.8%, 54.1% and 1.24%, respectively (Figure 1).

The area under the curve (AUC) was 0.729 (SE 0.059; 95% CI 0.613-0.826) for sCD146 and 0.588 (SE 0.066; 95% CI 0.468-0.870) for CIMT. ROC curves for sCD146 differed significantly in differentiating patients with type 1 diabetes from healthy controls (p = 0.0047) (Figure 1). A significantly higher percentage of patients than of controls had sCD146 levels >260 ng/ml (21/37 (56.8%) vs. 2/37 (5.4%), p = 0.00011).

## Discussion

The present study assessing the relationship between early atherosclerosis and endothelial dysfunction in type 1 diabetic patients showed that sCD146 levels were higher in patients than in healthy controls, whereas CIMT values did not differ in these two groups. An sCD146 concentration >260.74 ng/ml was 10.5-fold more likely in a subject with type 1 diabetes than in a healthy control subject, whereas a CIMT >0.5 mm was only 1.24-fold more likely in a diabetic individual, suggesting that sCD146 is a better predictor of early atherosclerosis than CIMT.

The greater ability of sCD146 than of CIMT in distinguishing between individuals with type 1 diabetes mellitus and healthy controls in our study population appears consistent with early vascular endothelial dysfunction and atherosclerotic thickening being two different stages of atherosclerosis [31,32]. Furthermore, CIMT and endothelial dysfunction were not correlated in patients with early stage diabetic retinopathy [31,32]. In contrast, sCD146 levels were correlated with the progression of diabetic retinopathy, suggesting that sCD146 is associated with significant endothelial damage [23,27]. Similarly, fluorescence microcopy showed that the number of CECs was increased in subjects with type 2 diabetes [24,26], which is not surprising since insulin resistance, hypertension, hyperlipidemia and increased age may lead to endothelial dysfunction. In addition, many of these patients received medications that could affect endothelial cell function, including statins, aspirin, antihypertensive drugs, and clopidogrel [33].

Although increased sCD146 as a marker of increased CECs predicting endothelial damage is an expected outcome in type 2 diabetics, type 1 diabetes is a pure hormone deficiency disease, providing a better basis for studying the impact of hyperglycemia per se on mechanisms of vascular injury. Thus, the increase in sCD146 levels in our population of patients with uncomplicated type 1 diabetes, with no additional cardiovascular risk factors, suggests that hyperglycemia, and possibly hypoinsulinemia, may play primary roles in the induction of endothelial damage. A previous study in patients with type 1 diabetes without concomitant disorders found an increased number of CECs by fluorescence microscopy [25]. Those patients, however, had a longer duration of diabetes than in our study, with many of those patients having microvascular complications such as retinopathy, nephropathy and neuropathy. Thus, the increase in sCD146 levels in those patients, relative to controls, was consistent with their concomitant microvascular complications. In contrast, our patient population did not have any diabetes related complications, including microalbuminuria, as well as having similar age, gender and lipid profiles as our healthy control groups. Thus, the higher sCD146 levels in our patient than in our control group may be a marker of early stage complications in patients with type 1 diabetes.

Our finding, that CIMT values were similar in the type 1 diabetes and control groups, seems to contrast with the results of The Epidemiology of Diabetes Intervention and Complications (EDIC) Study, which reported that CIMT was significantly higher in adults with type 1 than type 2 diabetes of both sexes at 6 years of follow up, suggesting the precocious development of atherosclerosis in patients with type 1 diabetes [34]. However, our patients were younger and had a shorter duration of diabetes than patients in the EDIC Study. Moreover, none of our patients had diabetes-related micro- and macrovascular complications or additional cardiovascular risk factors. Thus, our finding, that CIMT was similar in patients and controls, was not surprising. Moreover, although CIMT has been reported to be significantly higher in diabetic patients than in non-diabetic subjects, CIMT progression was reported to be due not only to diabetes per se, but to other concomitant metabolic abnormalities, in particular arterial hypertension [14]. Thus, our failure to detect diabetes-dependent changes in CIMT in our patients may be due to their lack of diabetes-related complications and additional cardiovascular risk factors.

Notably, mean CIMT level in our patients with type 1 diabetes was lower than in previous studies [35-40]. In this regard, given the theory of the pathogenesis of atherosclerosis [41], with detection of endothelial dysfunction predicting an early functional disturbance of the vessel wall and measurement of IMT functioning as an early morphological sign [42], our findings emphasize the likelihood that sCD146 is a more sensitive and earlier predictor of subclinical endothelial dysfunction than CIMT.

Likewise, endothelial dysfunction has been suggested to be detected very early in the life of insulin resistant subjects despite a lack of significant structural changes, indicated by a thickening of the intima-media layer [16]. Consistent with our finding that, compared to CIMT, sCD146 was better able to distinguish individuals with type 1 diabetes mellitus from healthy controls, markers of endothelial dysfunction have been regarded as more helpful than measuring the thickness of the vascular wall in identifying subjects at high risk for accelerated atherosclerosis at an early stage [16].

## Conclusions

In conclusion, our findings showed that sCD146 but not CIMT was significantly higher in a young population of individuals with type 1 diabetes, with a relatively short duration of diabetes and a lack of diabetes-related complications and additional cardiovascular risk factors, than in matched healthy controls. Moreover, sCD146 was better able to distinguish type 1 diabetes from healthy controls than was CIMT. Our results suggest that sCD146 levels may be a more sensitive marker than CIMT for the earlier identification of type 1 diabetic patients at high risk for atherosclerosis.

## Abbreviations

BMI: Body mass index; CECs: Circulating endothelial cells; CIMT: Carotid intima-media thickness; sCD146: Soluble CD146.

## Competing interests

The authors declare that they have no conflicts of interests.

## Authors’ contributions

AH designed the research, interpreted the data, and drafted and revised the manuscript; SCD drafted the manuscript; SCD and MA analyzed and interpreted the data; SDO measured CIMT; and SCD and MA provided intellectual content of critical importance to the work. AH had primary responsibility for the final content of the manuscript. All authors read and approved the final manuscript.
